# Urinary fatty acid biomarkers for prostate cancer detection

**DOI:** 10.1371/journal.pone.0297615

**Published:** 2024-02-09

**Authors:** Elizabeth Noriega Landa, George E. Quaye, Xiaogang Su, Sabur Badmos, Kiana L. Holbrook, Thomas J. Polascik, Eric S. Adams, Sriram Deivasigamani, Qin Gao, Michael H. Annabi, Ahsan Habib, Wen-Yee Lee

**Affiliations:** 1 Department of Chemistry and Biochemistry, University of Texas at El Paso, El Paso, Texas, United States of America; 2 Department of Mathematical Sciences, University of Texas at El Paso, El Paso, Texas, United States of America; 3 Department of Urological Surgery, Duke University Medical Center, Durham, North Carolina, United States of America; 4 Biologics Analytical Operations, Gilead Sciences Incorporated, Oceanside, California, United States of America; 5 Michael H Annabi MD PA, El Paso, Texas, United States of America; Université Clermont Auvergne - Faculté de Biologie, FRANCE

## Abstract

The lack of accuracy in the current prostate specific antigen (PSA) test for prostate cancer (PCa) screening causes around 60–75% of unnecessary prostate biopsies. Therefore, alternative diagnostic methods that have better accuracy and can prevent over-diagnosis of PCa are needed. Researchers have examined various potential biomarkers for PCa, and of those fatty acids (FAs) markers have received special attention due to their role in cancer metabolomics. It has been noted that PCa metabolism prefers FAs over glucose substrates for continued rapid proliferation. Hence, we proposed using a urinary FAs based model as a non-invasive alternative for PCa detection. Urine samples collected from 334 biopsy-designated PCa positive and 232 biopsy-designated PCa negative subjects were analyzed for FAs and lipid related compounds by stir bar sorptive extraction coupled with gas chromatography/mass spectrometry (SBSE-GC/MS). The dataset was split into the training (70%) and testing (30%) sets to develop and validate logit models and repeated for 100 runs of random data partitioning. Over the 100 runs, we confirmed the stability of the models and obtained optimal tuning parameters for developing the final FA based model. A PSA model using the values of the patients’ PSA test results was constructed with the same cohort for the purpose of comparing the performances of the FA model against PSA test. The FA final model selected 20 FAs and rendered an AUC of 0.71 (95% CI = 0.67–0.75, sensitivity = 0.48, and specificity = 0.83). In comparison, the PSA model performed with an AUC of 0.51 (95% CI = 0.46–0.66, sensitivity = 0.44, and specificity = 0.71). The study supports the potential use of urinary FAs as a stable and non-invasive alternative test for PCa diagnosis.

## Introduction

Prostate cancer (PCa) is the second most common type of cancer in males worldwide and is the most diagnosed cancer type in the USA [1, 2]. For 2023, it has been estimated that there are 288,300 new PCa cases and approximately 34,700 deaths caused by PCa in the USA alone [3]. As early detection is key to prevent PCa related deaths, clinicians often prescribe the prostate-specific antigen (PSA) test to men of 50 to 69 years old every 2 to 4 years [4]. Currently, the recommended screening test for early screening of PCa is the PSA test. This screening method measures the PSA levels in the blood, and elevated levels of PSA could be an indication of PCa. When PSA levels are high, patients are often referred to receive a prostate biopsy to verify the suspicion of a PCa diagnosis. However, PSA levels have been known to increase by other factors unrelated to PCa, which in turn causes a decrease in its specificity and accuracy [5–9]. About 60–75% of men who have a biopsy due to an elevated PSA do not have PCa [10, 11]; and a prostate biopsy is an invasive test that can cause rectal bleeding, discomfort, pain, or infection [12, 13]. All these issues of PSA highlight the clinical need for screening tools that can more accurately identify men who are most likely to benefit from early diagnosis and treatment while avoiding the over-diagnosis of clinically insignificant or low grade PCa.

A relatively new direction for PCa screening is using small-molecule biomarkers, i.e., metabolomics. Cancer metabolomics, which involves the characterization of metabolite profiles in cancer cells, can provide an accurate read-out of the physiology and biochemical activity of tumor cells [14, 15]. Hence, metabolomes can be considered as the final products of the overall molecular pathways in the other “omics”. Recently research has shown promising evidence using trained animals to detect scents in urine from PCa with sensitivities and specificities at 91 to 100% [16]. As the odor signature of urine is produced by volatile organic compounds (VOCs), those research findings support the research rationale that particular VOCs could be differentially produced by normal and cancer cells [17]. By using a technique like gas chromatography and mass spectrometry (GC-MS) the VOCs responsible for the ‘distinctive’ PCa smell can be identified and quantified [18–20] to explore their applications in cancer detection as well as the pathways that could be responsible for the production of those VOCs.

Among those urinary VOC biomarkers, lipid related compounds are of special interest. It is reported that altered lipid metabolism is an important marker of cancer, mainly because lipids are needed for rapid cell proliferation [21]. Lipids are required for signaling within cells, and are resources for membrane assembly, energy storage and production [22]. The source of the lipids appropriated by cancerous cells can come from *de novo* lipid synthesis or from exogenous lipids through lipolysis. Both *de novo* lipid synthesis and lipolysis are closely related to fatty acids (FAs) production in humans. Of the aforementioned mechanisms, lipid synthesis produces FAs from carbohydrate breakdown and lipolysis receives FAs products from triglyceride breakdown [23, 24]. FAs can undergo esterification to produce phospholipids, which are then used by malignant cells for functions like cell membrane synthesis, migration, transduction, etc. [25, 26]. In a healthy subject the FAs used for these same functions are obtained mainly from diet. However, in the context of malignant cells, there is an increase in FA uptake from de novo synthesis and diet in order to meet the increasing need of lipid components [27–29]. Unlike most cancer types, PCa shows a preference for FAs as the source of energy component over glucose breakdown to uphold the rapid proliferation characteristic of malignant cells [30–32]. Research found that alterations in lipid metabolism can lead to changes in the fatty acid composition of cells and tissues in PCa. Watt et al. [33] reported that suppression of fatty acid intake in mice had slowed PCa progression and reduced oncogenic lipid signaling. Recent research has also suggested that FAs may also serve as potential biomarkers for prostate cancer [34–36].

Our previous work has shown that urinary VOCs can be used for PCa diagnosis [17]. As many of the significant VOCs are involved in lipid related pathways, this study aimed to use lipid related compounds in urine, such as FAs, fatty acid methyl esters, sterols, and lipid derivatives, for a PCa diagnostic model. The performance of the FA model was then compared with the diagnosis outcome based on PSA to evaluate the clinical applicability in PCa diagnosis.

## Materials and methods

### Patients and controls

Internal Review Board (IRB) approval (University of Texas at El Paso (UTEP) IRB 836503–9) for the study was obtained prior to the study. Shelved and de-identified urine specimens were obtained from patients present at the Duke University Medical Center, Durham, North Carolina; Eastern Virginia Medical Center, Norfolk, Virginia; Michael H. Annabi Internal Medicine Clinic, El Paso, Texas, and Massachusetts General Hospital, Boston, Massachusetts. Only de-identified information (such as age, race, and pathology outcomes) will be used to indicate that the samples came from a participant positive or negative for PCa. No information on diet factors, such as smoking and drinking, for the patients was made available for this study.

All participants in the study were men subjected to a PSA blood test and their cancer status was designated by subsequent prostate biopsy. A total of 566 participants were included, of which 334 were PCa biopsy-designated positive patients (hereafter referred as PCa or PCa positive) and 232 were PCa biopsy-designated negative control patients (hereafter referred as control). The detailed demographics of the patient samples are summarized in Table 1.

**Table 1:** Demographic information for the study cohorts.

	N	Age	PSA (ng/mL)
**PCa**	334	66 (43–91)	3.8 (0.01–1987)
**Control**	232	70 (32–94)	2.2 (0.1–28)
***P* value**			0.123^1^
**Gleason Grade**			
**Group 1**	180 (53.9%)		
**Group 2 and 3**	120 (35.9%)		
**Group 4**	16 (4.8%)		
**Group 5**	18 (5.4%)		

Continuous variables are presented as median (interquartile range). Categorical variables presented as n (%).

^1^ P value obtained from Wilcoxon test of the PSA between prostate cancer and control groups.

### Chemicals

Mirex (99.0%, Dr. Ehrenstorfer GmbH, Germany) was selected as the internal standard and a 1 mg/L (1 ppm) solution was prepared in methanol for analysis. Hydrochloric acid (HCl) (37%) was purchased from Sigma-Aldrich (St. Louis, MO) and a 2M solution was prepared in deionized (DI) water. HPLC grade Water (Sigma-Aldrich) was used for sample preparation.

### Stir bar sorptive extraction and gas chromatography/mass spectrometry analysis

The method for urine analysis was established by Gao et.al [17]. Briefly, 1.0 mL of urine was added to an amber glass vial containing 19.0 mL of HPLC grade water, 300 μL of Mirex (1 ppm), and 600 μL of HCl (2M). To each glass vials, 1 stir bar (Twister, 10 mm 1 mm, Gerstel, Mülheim and der Ruhr, Germany) coated with polydimethylsiloxanes was added and the mixture was stirred at 1,000 rpm for 2 hours. Then the stir bar was removed from the solution and rinsed with DI water, dried with lint-free paper (Kimtech Science™ Kimwipes™ Delicate Task Wipes) and placed into a thermal desorption tube (TDT, Gerstel) for analysis.

Chemical analysis prior to 2020 were performed on a thermal desorption unit (TDU, Gerstel), coupled with Gas Chromatography/Mass Spectrometry (6890/5973-N GC/MS, Agilent Technologies, Wilmington DE). The GC/MS was equipped with a ZB-5ms capillary column (30 m X 0.25 μm X 0.25 μm; Phenomenex, Torrence, CA). From 2020 and onwards, chemical analysis was performed in a thermal desorption unit (TD3.5+, Gerstel) coupled with an 8890/5977B-N GC/MS (Agilent Technologies, Wilmington DE). The GC/MS was equipped with a HP-5ms Ultra Inert capillary column (30 m X 0.25 μm X 0.25 μm; Agilent J&W Columns). The thermal desorption, GC and MS programs were kept consistent on both instruments.

The thermal desorption process was set under a splitless mode and programmed as follows. The initial temperature was set at 45°C and held for 0.5 minutes; the temperature was ramped at 60°C/minute to 300°C and held for 5 minutes. Desorption gas flow was set at 1.0 mL/min. During desorption, the compounds were concentrated in a cold injection system (CIS4, Gerstel) at -40°C. Once the desorption process was completed, the CIS4 was heated to 300°C at 12°C/s and held for 5 minutes. The GC oven temperature was programmed as follows. The temperature was initially set at 35°C and held for 5 minutes, ramped to 300°C at 10°C/min and held for 10 minutes. The urinary VOCs were detected by mass selective detector in scan mode (20–500 m/z) and identified by the National Institute of Standards and Technology Library (NIST17).

### Statistical analysis

The urinary VOCs were identified by the library NIST17 according to the matching quality of the MS spectra produced by the instrument. We implemented a filter to a matching quality of 50% or greater to ensure satisfactory VOC identification in further data processing. The internal standard, Mirex, was used to determine relative response for the identified VOCs and for statistical analysis.

From the 566 samples analyzed, 21,547 VOCs were identified and formed the initial data set. The resultant data set indicates a fairly zero-inflated and ultra-high dimensional (p>>n) modeling problem. This observation leads to using the Wilcoxon Rank Sum Test, a univariate screening approach that makes no parametric assumptions about the response variable, for the initial variable screening [37]. After the Wilcoxon Rank Sum test, a total of 1,279 significant VOCs (p < 0.05) were subjected to an over-representation analysis through the online resource Consensus PathDB (CPDB, http://cpdb.molgen.mpg.de/). This analysis applies the hypergeometric test, where a p value is assigned. The results from this test provided the possible pathways were related to the VOCs found to be statistically significant between PCa patients and control patients.

**Fig 1:**
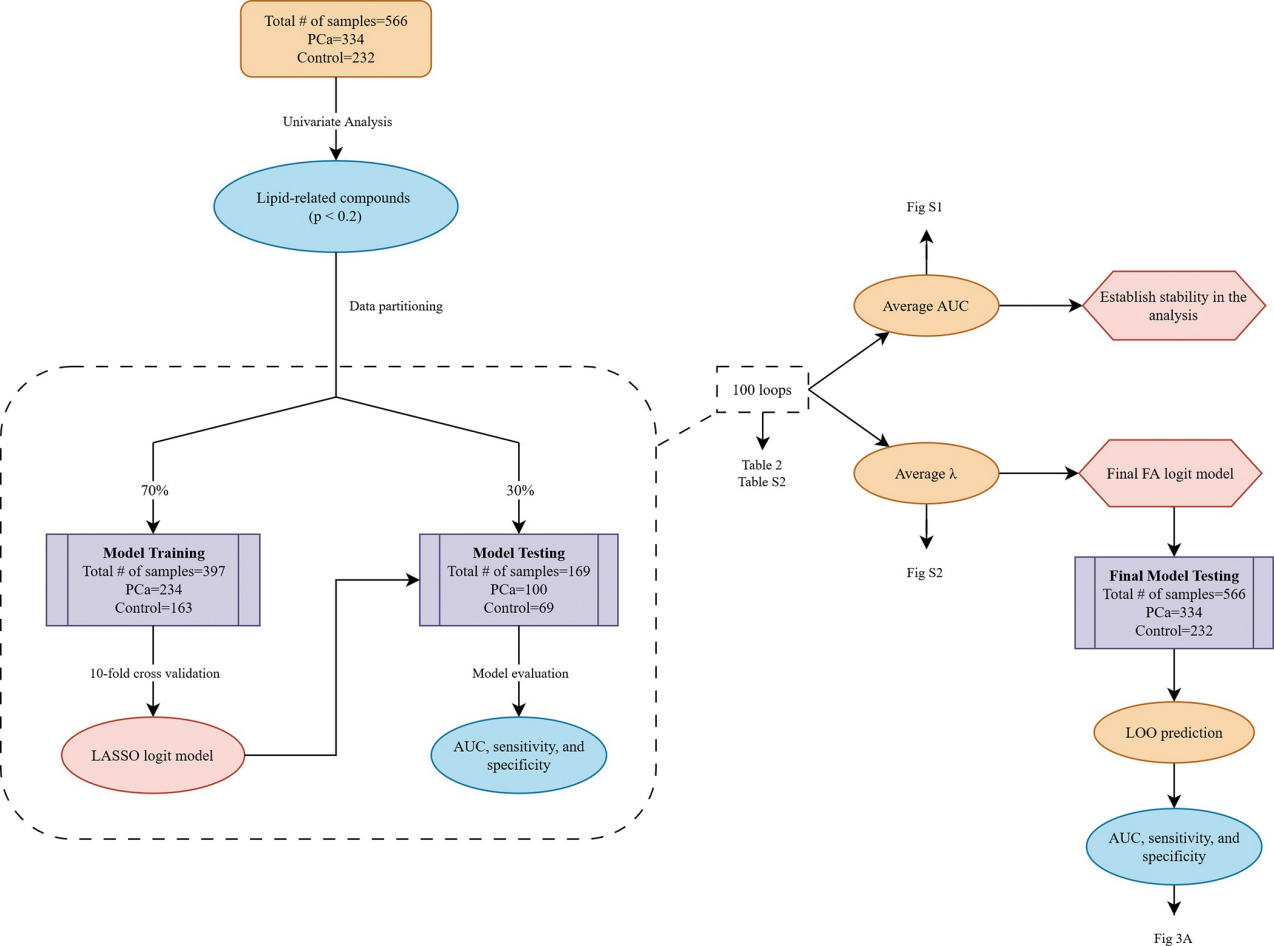
Process for creating the final FA diagnostic model. The entire cohort (n = 566) randomly split to training (70%) and testing (30%) groups. This process was repeated over a 100 loop to stablish stability in the analysis and to obtain the optimal tuning parameter (λ) which was used to create the final FA logistic regression (logit) model. The final FA logit model was tested on the full cohort and evaluated by AUC via Leave-One-Out (LOO) prediction.

The pool of 21,547 VOCs was screened using keywords (“-ic acid”, “ester”, “-ate”, “chol-“, and “phos-“) to filter out lipids, fatty acids, and their derivates to continue to the next step (henceforth they will all be referred to as FAs). Based on the Wilcoxon test, a liberal cutoff of p < 0.2 was applied to select a pool of significant FAs for the development of a logistic regression model with LASSO penalty. For model development, 566 samples were divided into the training set and testing set in a ratio of 70:30 (Fig 1). The training set, via 10-fold cross-validation, was used to select the optimal tuning parameter (λ) for the LASSO logistic regression. This approach allowed us to find the optimal set of hyperparameters that resulted in the best performance for the model. By applying LASSO regularization to the logistic regression, the model could be improved in terms of variable selection, interpretability and generalizability [38, 39]. The testing set was then used to evaluate the performance of the model via the receiver operating characteristics (ROC) plot and the area under the curve (AUC). The analysis was done for 100 runs to acquire 100 AUC and the optimal tuning parameter (λ) values. The loop serves two purposes, one was to assess the model’s generalization performance and variability due to the random sampling of the data. The second purpose was to obtain the optimal λ from the loop to create a final diagnostic model. The final model, which included non-zero coefficients after applying LASSO regularization, was evaluated using leave-one-out (LOO) prediction. The optimal cutoff point for the confusion matrix was determined using the Youden Index [40]. At this optimal cutoff, we extracted the corresponding sensitivity and specificity values, which provided essential information about the model’s performance in correctly identifying true positives and true negatives, respectively.

A logistic regression model was built separately using the PSA values as the sole predicting variable. The same validation procedure, i.e., LOO prediction, was applied to this PSA model as that used in the final FA model. The comparison between the PSA and FA final model included evaluation metrics, such as the ROC, AUC, specificity, and sensitivity. All statistical analyses were carried out in RStudio.

## Results

**Fig 2:**
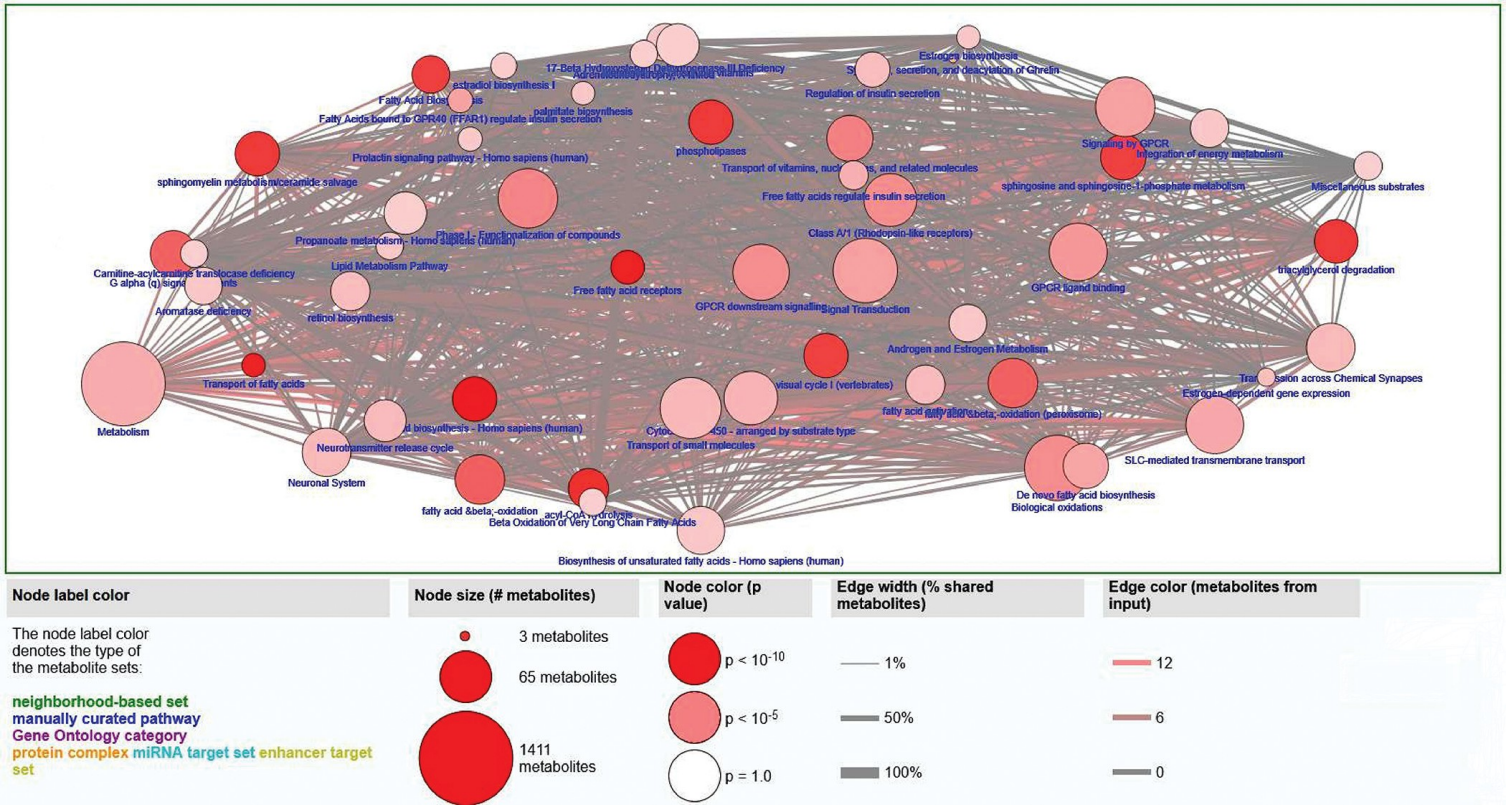
Over-representation analysis of significant VOCs in PCa. This plot represents the relationships between the significant VOCs found in urine and their relevant pathways. Each node in the plot represents a pathway; the size corresponds to the number of metabolites involved in the pathway, and the color intensity corresponds to the p value generated by the hypergeometric test.

The study utilized SBSE-GC/MS to extract and analyze potential lipid biomarkers for PCa diagnosis in 566 urine samples. The library NIST17 identified a total of 21,547 VOCs in the pool of samples. Univariate analysis found that 1,279 VOCs were significant (p < 0.05). The entire list of significant VOCs was subjected to an over-representation analysis through the online resource CPDB to find pathways related to the pool provided. CPDB identified 52 pathways related to the significant VOCs, and it was found that many of the pathways identified were lipid and fatty acid related. As shown in Fig 2, the over-representation analysis highlighted multiple lipid related pathways, which are represented by nodes in the plot.

Based on the pathway analysis, lipid and fatty acid pathways (S1 Table in the Supplementary Materials) were predominant pathways that involved the 1,279 significant VOCs. We then further focused on using FAs and lipids-related compounds in the study. Among the 21,547 VOCs, 618 FAs and lipids were identified. Univariate screening found 60 significant (p < 0.05) FAs and lipids in the VOC pool. However, a liberal cutoff (p < 0.2) was implemented to select significant FAs for model development and rendered 112 FAs that were used to create a LASSO logistic regression model. To develop the diagnostic model, the entire dataset of 566 patients underwent random data partitioning (70:30). The training cohort made up of 397 (PCa = 234, control = 163) urine samples, was used to develop a diagnostic model. The model was tested on the training data (n = 169; PCa = 100 and control = 69) and evaluated by ROC and AUC. This process was repeated over a 100 loop (Fig 1) with the purpose of establishing stability over the analysis (S1 Fig) and extracting the optimal tuning parameters (S2 Fig) to create a final diagnostic model. The performance of the 100 runs was listed in S2 Table in the Supplementary Materials, and Table 2 summarizes the overall performance throughout the loop. The results supported the stability of the FA model.

**Table 2:** Summarized results from the 100 loops.

	Cutoff Point	Sensitivity	Specificity	AUC	Optimal λ
**Average**	0.564	0.662	0.581	0.685	0.028
**Standard deviation**	0.020	0.118	0.107	0.036	0.013

From the loop we obtained the average optimal tuning parameter, λ (Table 2), which was used to create a final LASSO logistic regression FA model. The optimal λ selected 20 FAs for the final model (Table 3 and S3 Table). It is noteworthy that even though we originally included lipid-related compounds like cholesterol as potential variables, only fatty acids and FA related compounds were selected in the final model. Henceforth we will refer to the final diagnostic model as the FA model. The selected FAs in the diagnostic model were predominant evenly in either PCa or control group. Furthermore, these FAs covered a wide range of carbon chain lengths, from short (C5 or less) to very long (C22).

**Table 3:** Significant FA for prostate cancer diagnostic model sorted by the chain length.

FA Chain	Chemical Name	Chemical Formula	P value^1^	Dominating Group
C4:1	Tiglic acid	C_5_H_8_O_2_	1.07E-09	PCa
C5:1	4-Pentenoic acid, 2-methyl-, isobutyl ester	C_10_H_18_O_2_	0.011	Control
C6:0	Hexanoic acid, 3-tetradecyl ester	C_20_H_40_O_2_	0.032	Control
C9:0	Nonanoic acid	C_9_H_18_O_2_	5.38E-06	PCa
C10:0	n-Decanoic acid	C_10_H_20_O_2_	4.76E-08	PCa
C11:0	Undecanoic acid, 11-bromo-, undecyl ester	C_12_H_23_BrO_2_	1.22E-03	PCa
C13:0	Tridecanoic acid	C_13_H_26_O_2_	1.33E-05	PCa
C14:0	Tetradecanoic acid	C_14_H_28_O_2_	3.71E-04	Control
C15:0	i-Propyl 14-methyl-pentadecanoate	C_19_H_38_O_2_	1.56E-04	PCa
C15:0	Pentadecanoic acid, 14-methyl-, methyl ester	C_17_H_34_O_2_	0.023	PCa
C16:0	Methyl 10-methyl-hexadecanoate	C_18_H_36_O_2_	0.029	Control
C17:0	Heptadecanoic acid	C_17_H_34_O_2_	1.14E-03	Control
C18:0	Methyl 2-hydroxystearate, TMS derivative	C_22_H_46_O_3_Si	0.004	PCa
C18:0	Stearic acid hydrazide	C_18_H_38_N_2_O	0.035	PCa
C18:0	9-Octadecenoic acid	C_18_H_34_O_2_	1.45E-05	Control
C18:1	9-Octadecenoic acid, (E)-	C_18_H_34_O_2_	3.82E-04	Control
C18:1	9-Octadecenoic acid (Z)-, methyl ester	C_19_H_36_O_2_	6.87E-04	Control
C18:1	cis-9-Octadecenoic acid, propyl ester	C_21_H_40_O_2_	0.007	Control
C18:1	trans-13-Octadecenoic acid, methyl ester	C_19_H_36_O_2_	0.041	Control
C22:0	Docosanoic acid, docosyl ester	C_44_H_88_O_2_	2.59E-04	PCa

FA chain column presented as Carbon chain length: number of double bonds.

^1^P value obtained from Wilcoxon test of the FA ratio between prostate cancer and control groups.

**Fig 3:**
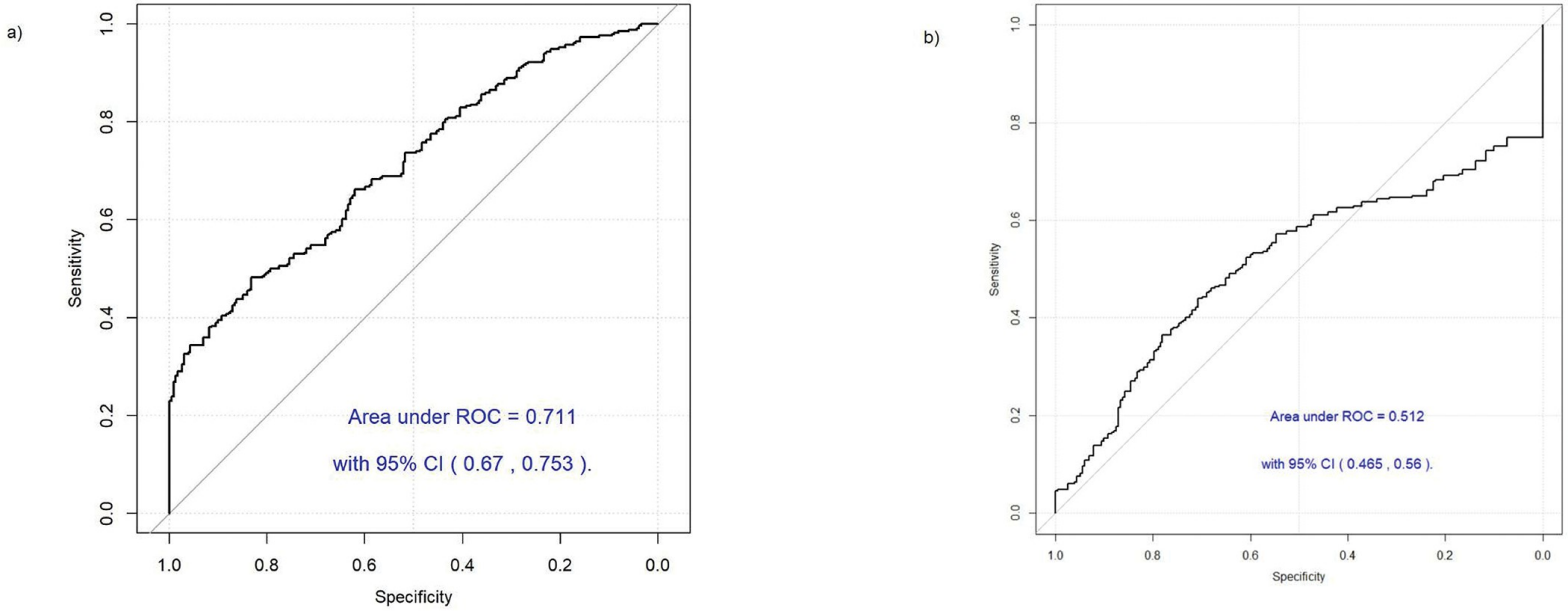
Performance of the diagnostic models for PCa diagnosis. a) Performance of final FA diagnostic model. b) Performance of PSA diagnostic model. Receiver Operating Characteristic (ROC), Confidence Interval (CI), Area under the curve (AUC).

The final FA model was tested on the entire dataset (n = 566) and evaluated by AUC via Leave-one-out (LOO) prediction. The performance of the FA model (Fig 3A) had an AUC of 0.711 (95% CI = 0.670–0.753), sensitivity of 0.48, and specificity of 0.83 at the optimal cutoff point of 0.58. As a comparison, the diagnostic performance using PSA was tested. The PSA model performed (Fig 3B) with an AUC of 0.512 (95% CI = 0.465–0.560), sensitivity of 0.44, and specificity of 0.71 at a cutoff point of 0.58. The side-by-side comparison of the performance of the FA and PSA models can be seen in Table 4, indicating that the FA model outperformed the PSA model.

**Table 4:** Performance of prostate cancer diagnostic models.

	FA diagnostic model	PSA diagnostic model
**Sensitivity**	0.48	0.44
**Specificity**	0.83	0.71
**AUC**	0.71	0.51
**95% CI** ^1^	0.67–0.75	0.46–0.56

Area Under the Curve (AUC), Prostate Specific Antigen (PSA), Confidence Interval (CI).

^1^95% CI: 95% confidence interval in reference to AUC.

## Discussion

It has been suggested that understanding the changes in fatty acid metabolism in prostate cancer could provide direction for development of treatment or diagnosis [41]. Through the results of the over-representation analysis, the significant VOCs found in PCa patients urine samples were related to lipid or fatty acid pathways (S1 Table), such as fatty acid β-oxidation, de novo fatty acid biosynthesis, palmitate biosynthesis, etc. The findings support the reported studies [42, 43] indicating that lipid metabolism is strongly linked to PCa. Thus, FAs and lipids could be cancer biomarkers to develop a better diagnostic model than the PSA test for PCa. It should be noted that due to the lack of details on patient diet, the origin of FA found in urine is unknown and out of the scope of this present study. We cannot claim that the FA found in the urine from patients were synthesized in the body or originate from diet, but we did find a significant (p < 0.05) correlation of FAs to PCa status. We used the evaluation metrics such as AUC obtained from the ROC analysis, as well as the sensitivity and specificity. This evaluation method allowed us to assess the model’s performance and discrimination ability effectively, accounting for the impact of each observation individually while leaving it out during the prediction [44]. As shown in Table 4, the FA model demonstrated higher sensitivity, specificity, and overall higher accuracy than the PSA model for PCa diagnosis.

Although the study of the pathways involving the FAs selected by the FA model was beyond the scope of this study, we incorporated literature to formulate implications on their relevance and possible functions. Extra attention was paid to carbon chain length and the saturation of the fatty acids selected in the final FA model because it has been observed that genes involved in fatty acid elongation are upregulated in PCa patients [45]. It has also been observed that the degree of saturation in the fatty acid chain also has different effects on PCa. For instance, polyunsaturated fatty acids have been found to suppress PCa cell proliferation [46]. In general, the FAs in the diagnostic model cover a broad range of carbon chain lengths and are mostly fully saturated fatty acid chains (Table 3 and S3 Table).

In the case of short-chain FA, which are FAs with C5 or less, our FA model had one C4 FA that was found predominantly in PCa patients and one C5 found predominantly in the control group respectively. The FA that was selected with the highest frequency during the loop was tiglic acid (C4:1). The secretion of tiglic acid could be the consequence of multiple carboxylase deficiency, characterized by activity deficiency of biotin-related enzymes [47]. One of the enzymes affected is pyruvate carboxylase, which supports the Warburg effect by supplying glucose derived from pyruvate through the Krebs cycle. The Warburg effect states that cancer cells prefer glucose as their main energy source, but as discussed previously, PCa prefers fatty acids as an energy source. We therefore hypothesized that tiglic acid could be an indicator of inhibition of pyruvate carboxylase and this could be an indication of PCa turning to an alternative energy source instead of using glucose [48, 49]. In addition, tiglic acid has a similar structure to butyric acid (C4:0), which has been found to promote apoptosis in colorectal cancer cells [50]. The other short-chain FA in our model was 4-pentenoic acid, 2-methyl-, isobutyl ester (C5:1), which is similar in length to valeric acid (C5:0). Valeric acid has been found to inhibit histone deacetylase enzymes (HDAC) which are overexpressed in multiple cancers [51]. Recently, valeric acid has been reported to prevent liver cancer progression through inhibition of histone deacetylase [52]. The predominant occurrence of 4-pentenoic acid and 2-methyl-, isobutyl ester in the control group could be due to their structural similarity to valeric acid causing potential inhibition of HDAC1 levels [53].

Our diagnostic model contained 4 types of medium-chain FAs, which encompass chain lengths from C6 to C12. Hexanoic acid 3-tetradecyl ester (C6:0) selected in the FA model was found to be dominant in the control group. This finding was in agreement with the report by Narayanan et al. [54], who reported that hexanoic acid reduced cell viability significantly (p < 0.05) in human skin, colorectal, and breast cancer cell lines. The other medium-chain FAs included in the model, C11, C10, and C9, were found predominantly in PCa patients. Uchiyama et al. [55] reported that FA chains of C8 and C10 showed a significant (p < 0.01) correlation between colorectal cancer stages. Nonanoic acid (C9:0) is fatty acid that is also a naturally occurring component, it has been found to be significant (p = 0.011) between lung cancer and control patients [56]. As nonanoic acid can be ingested and was found mainly in PCa patients, it would support the claim that exogenous fatty acids are preferred by malignant cells for rapid proliferation [57]. A recent study [58] identified undecanoic acid (C11:0) as a strong inhibitor of cancer cell proliferation; however, it contradicts with our findings.

Most of the fatty acids in our diagnostic model were long-chain fatty acids, from C13 to C18. Tridecanoic acid (C13:0) was able to detect lung cancer screening with an AUC 0.81 [59]. Tetradecanoic acid (C14:0) can be found in nutmeg, and we found it predominantly in the control group. While there are no previous studies on how tetradecanoic acid is linked to PCa, there have been previous reports of its branched derivatives, i.e. 12-methyltetradecanoic acid inducing apoptosis in PC3 prostate cancer cell lines [60]. Another FA, Pentadecanoic acid (C15:0) and one of its derivates were included in the FA model predominantly in PCa patients. Research showed that pentadecanoic acid suppressed the invasiveness of malignant breast cancer cell lines through the inhibition of Janus kinase 2/signal transduced and activator of transcription 3 (JAK2/STAT3) [61]. However, in PCa the JAK2/STAT3 signaling pathway can promote chemoresistance by enhancing regulators of PCa progression [62]. The presence of two variations of pentadecanoic acid in the PCa group appeared to indicate that they could promote PCa progression through the JAK2/STAT3 pathway. Another long-chain FA, methyl 10-methyl-hexadecanoate (C16:0) was found predominately in the control group. The finding supported the effect of palmitic acid (C16:0) and its relation to suppress PCa cell growth though inhibition of the PI3K-AKT-mTOR pathway [63]. Additionally Kim et al. [64] observed that heptadecanoic acid (C17:0) had anti-proliferative properties against pancreatic cancer cell lines, and this effect was also present in the case of malignant cell lines resistant to chemotherapeutic agents. Heptadecanoic acid has been found to exhibit these anti-cancer effect on lung cancer cell lines through suppression of the PI3K-AKT-mTOR pathway [65]. Since many FAs selected by our model have links to suppression of the PI3K-AKT-mTOR, we hypothesized that these FAs could have a role in the prevention of loss function of PTEN, a lipid/protein phosphatase. which has been identified to negatively regulate PI3K-AKT-mTOR signaling in PCa [66].

There were 7 compounds in the FA model containing a C18 chain, which have been identified as a recurring fatty acids as PCa biomarkers [67–71]. Snider et al. [72] found that a 5-lipid model to determine PCa aggressiveness had an AUC 0.882 (CI = 0.803–0.954), of which all 5 lipids had at least one 18 carbon chain. That five-lipid model was composed of sphingomyelin (18:0/24:1), trihexosylceramide (18:1/16:0), tetrahexosylceramide (18:1/16:0), ceramide (18:1/22:0), and ceramide (18:1/24:1). Skotland et al. [73] reported that they were able to distinguish between PCa patients and control with a 93% sensitivity and 100% specificity with a 3 lipid model using phosphatidylserine (18:1/18:1), lactisylceramide (18:1/16:0), and phosphatidylserine (18:0/18:2). In our model, six of C18 FAs were found predominantly in the control group and had fatty acid structure similar to oleic acid (C18:1). This similarity could explain their prevalence in the control group as oleic acid is known to induce apoptosis to malignant cells by inhibiting the phosphatidylinositol-3-kinase protein kinase B and mammalian target of rapamycin (PI3K-AKT-mTOR) pathway [74].

In our FA model, there was only one very-long chained FA, Docosanoic acid (C22:0) selected and dominant in PCa group. Docosanoic acid was found to be upregulated in an animal model and linked to gastric cancer metastasis [75], which supports our finding of it predominantly in the PCa group.

## Conclusions

This study applied a solventless extraction method for a high throughput fatty acid analysis in urine. The FA diagnostic model was proven to be stable over 100 runs. It was also shown to have a better performance than the PSA model in differentiating between PCa and control groups. The FA model for PCa diagnosis presents an alternative to the existing PAS method and the results support the continuing research using fatty acids as potential PCa biomarkers. Many FAs included in our diagnostic model present potential biological significance in cancer.

This study presents some limitations. Firstly, there is a possibility that some patients with prostate cancer may have been incorrectly categorized into the control group if their disease was undetected by prostate biopsy. Secondly, given the heterogeneous nature of PCa, the urinary FA diagnostic model developed in this study is expected to enhance its accuracy as the sample size is expanded. Thirdly, the impact of variables such as the timing of urine collection, dietary habits (such as smoking and drinking), individual disease risk, genetic predisposition, and environmental exposures on the measured FA concentrations remains unknown. These aspects will be a focus of future research investigations. Additionally, future work into the biological roles of fatty acids could be expanded to understand how their functions affect PCa progression.

## Supporting information

S1 Table Results from over-representation analysis. The table presents the results from performing an over-representation analysis with the significant (p < 0.05) VOCs.
(PDF)

S2 Table Results from the 100 loop. The table presents the results from performing the 100 loops of FA models.
(PDF)

S3 Table FA selected in the final PCa diagnosis model. The table presents the significant FA that were used to construct the diagnostic model, presented in order by their significance.
(PDF)

S1 Fig Histogram of the performance of the FA models in the 100 loop. The figure is a distribution of the performance (AUC) of the logistic regression models throughout the loop. The vertical line represents the mean (AUC = 0.685).
(PDF)

S2 Fig Histogram of the optimal tuning parameters λ in the 100 loop. The figure is a distribution of the optimal tuning parameters for the logistic regression models throughout the loop. The vertical line represents the mean (λ = 0.028).
(PDF)
