# Supplementary material for: Urinary fatty acid biomarkers for prostate cancer detection
Source: PLoS One. 2024 Feb 9;19(2):e0297615. doi: 10.1371/journal.pone.0297615 (PMC10857612; doi:10.1371/journal.pone.0297615)
Supplement: S1 Table — The table presents the results from performing an over-representation analysis with the significant (p < 0.05) VOCs. (PDF) [file pone.0297615.s001.pdf]

S1 Table. Results from over-representation analysis. The table presents the results from performing an over-representation analysis with the significant ($p < 0.05$) VOCs.

Pathway Name	Candidates Contained	P value[1]
Fatty acid biosynthesis-Homo sapiens (human)	7 (58.3%)	1.04E-11
Free fatty acid receptors	8 (33.3%)	8.16E-11
Transport of fatty acids	6 (66.7%)	1.18E-10
Acyl-CoA hydrolysis	8 (26.7%)	6.19E-10
Triacylglycerol degradation	8 (23.5%)	1.86E-09
Phospholipases	8 (23.5%)	1.86E-09
Sphingomyelin metabolism/ceramide salvage	8 (23.5%)	1.86E-09
Fatty acid biosynthesis	7 (29.2%)	4.14E-09
Sphingosine and sphingosine-1-phosphate metabolism	8 (21.1%)	4.85E-09
The visual cycle I (vertebrates)	8 (21.1%)	4.85E-09
Fatty acid β-oxidation	8 (14.0%)	1.41E-07
G alpha (q) signaling events	8 (13.8%)	1.62E-07
Fatty acid β-oxidation (peroxisome)	8 (13.8%)	1.62E-07
Transport of vitamins, nucleosides, and related molecules	7 (11.3%)	4.32E-06
Phase I - Functionalization of compounds	10 (6.3%)	6.52E-06
Biological oxidations	12 (4.7%)	1.46E-05
Class A/1 (Rhodopsin-like receptors)	8 (7.6%)	1.62E-05
GPCR downstream signaling	9 (6.5%)	1.65E-05
GPCR ligand binding	9 (5.8%)	4.20E-05
Signal Transduction	11 (4.2%)	9.32E-05
Signaling by GPCR	9 (5.2%)	9.97E-05
Fatty acids bound to GPR40 (FFAR1) regulate insulin secretion	3 (33.3%)	1.10E-04
De novo fatty acid biosynthesis	4 (16.0%)	1.54E-04
SLC-mediated transmembrane transport	8 (5.3%)	2.33E-04
Metabolism	20 (2.3%)	3.32E-04
Synthesis, secretion, and deacylation of Ghrelin	2 (66.7%)	3.80E-04
Free fatty acids regulate insulin secretion	3 (21.4%)	4.60E-04
Cytochrome P450 - arranged by substrate type	6 (6.1%)	7.12E-04
Transport of small molecules	8 (4.2%)	1.02E-03
Transmission across chemical synapses	5 (7.0%)	1.11E-03
Neuronal System	5 (7.0%)	1.11E-03
Neurotransmitter release cycle	4 (9.5%)	1.19E-03
Fatty acid activation	3 (15.0%)	1.37E-03
Retinol biosynthesis	3 (14.3%)	1.59E-03
Regulation of insulin secretion	3 (13.6%)	1.83E-03
Estrogen-dependent gene expression	2 (33.3%)	1.86E-03
Biosynthesis of unsaturated fatty acids - Homo sapiens (human)	3 (10.3%)	4.10E-03
Lipid Metabolism Pathway	2 (22.2%)	4.36E-03
Estrogen biosynthesis	2 (22.2%)	4.36E-03

Palmitate biosynthesis	2 (22.2%)	4.36E-03
17-Beta Hydroxysteroid Dehydrogenase III deficiency	3 (10.0%)	4.52E-03
Androgen and estrogen metabolism	3 (10.0%)	4.52E-03
Aromatase deficiency	3 (10.0%)	4.52E-03
Integration of energy metabolism	3 (10.0%)	4.52E-03
Prolactin signaling pathway - Homo sapiens (human)	2 (20.0%)	5.41E-03
Estradiol biosynthesis I	2 (18.2%)	6.57E-03
Beta oxidation of very long chain fatty acids	2 (16.7%)	7.83E-03
Adrenoleukodystrophy, X-linked	2 (16.7%)	7.83E-03
Carnitine-acylcarnitine translocase deficiency	2 (16.7%)	7.83E-03
Propanoate metabolism - Homo sapiens (human)	3 (7.9%)	8.83E-03
Metabolism of fat-soluble vitamins	3 (7.9%)	8.83E-03
Miscellaneous substrates	2 (15.4%)	9.18E-03

[1]P value obtained from over-representation analysis in ConsensusPathDB.
