# Supplementary material for: Urinary fatty acid biomarkers for prostate cancer detection
Source: PLoS One. 2024 Feb 9;19(2):e0297615. doi: 10.1371/journal.pone.0297615 (PMC10857612; doi:10.1371/journal.pone.0297615)
Supplement: S2 Table — The table presents the results from performing the 100 loops of FA models. (PDF) [file pone.0297615.s002.pdf]

S2 Table. Results from the 100 loop. The table presents the results from performing the 100 loops of FA models.

Loop #	AUC	Accuracy	Sensitivity	Specificity	Cutoff Point	Optimal λ
1	0.704	0.629	0.743	0.542	0.563	0.029
2	0.647	0.618	0.450	0.709	0.532	0.019
3	0.687	0.600	0.625	0.585	0.540	0.036
4	0.694	0.641	0.681	0.612	0.558	0.018
5	0.695	0.671	0.618	0.706	0.558	0.024
6	0.620	0.594	0.722	0.484	0.595	0.069
7	0.689	0.594	0.384	0.753	0.570	0.026
8	0.699	0.629	0.636	0.625	0.555	0.023
9	0.693	0.600	0.640	0.568	0.572	0.025
10	0.670	0.606	0.758	0.510	0.565	0.015
11	0.673	0.600	0.559	0.627	0.530	0.020
12	0.756	0.653	0.817	0.535	0.569	0.027
13	0.673	0.588	0.588	0.588	0.548	0.026
14	0.722	0.629	0.692	0.590	0.534	0.020
15	0.711	0.641	0.507	0.742	0.560	0.015
16	0.657	0.600	0.710	0.525	0.549	0.044
17	0.721	0.659	0.740	0.598	0.556	0.020
18	0.657	0.606	0.824	0.438	0.585	0.059
19	0.701	0.606	0.761	0.495	0.572	0.023
20	0.717	0.665	0.639	0.690	0.600	0.021
21	0.673	0.641	0.625	0.653	0.570	0.030
22	0.719	0.671	0.618	0.706	0.558	0.016
23	0.697	0.629	0.700	0.580	0.554	0.011
24	0.651	0.576	0.857	0.380	0.584	0.039
25	0.712	0.629	0.636	0.625	0.544	0.024
26	0.675	0.635	0.635	0.636	0.542	0.025
27	0.661	0.576	0.707	0.474	0.578	0.016
28	0.741	0.624	0.557	0.670	0.545	0.022
29	0.707	0.629	0.609	0.644	0.553	0.029
30	0.666	0.571	0.753	0.419	0.571	0.041
31	0.727	0.635	0.671	0.610	0.550	0.025
32	0.671	0.629	0.576	0.663	0.553	0.009
33	0.690	0.594	0.625	0.571	0.552	0.014
34	0.674	0.571	0.736	0.449	0.562	0.030
35	0.721	0.641	0.667	0.622	0.551	0.025
36	0.657	0.576	0.574	0.578	0.532	0.014
37	0.709	0.612	0.855	0.415	0.590	0.026
38	0.722	0.665	0.573	0.737	0.564	0.037
39	0.600	0.535	0.694	0.418	0.581	0.066

40	0.654	0.576	0.382	0.734	0.570	0.020
41	0.686	0.629	0.763	0.521	0.574	0.021
42	0.644	0.565	0.500	0.615	0.565	0.030
43	0.700	0.612	0.789	0.522	0.518	0.022
44	0.762	0.624	0.846	0.486	0.557	0.021
45	0.749	0.676	0.500	0.794	0.552	0.022
46	0.682	0.641	0.600	0.667	0.548	0.017
47	0.686	0.647	0.836	0.524	0.553	0.051
48	0.679	0.641	0.600	0.674	0.575	0.034
49	0.692	0.653	0.586	0.700	0.565	0.023
50	0.698	0.624	0.746	0.535	0.581	0.008
51	0.641	0.565	0.671	0.479	0.585	0.021
52	0.685	0.600	0.758	0.500	0.557	0.055
53	0.652	0.547	0.716	0.417	0.546	0.019
54	0.578	0.500	0.527	0.479	0.563	0.040
55	0.627	0.576	0.551	0.594	0.545	0.033
56	0.666	0.635	0.554	0.698	0.585	0.045
57	0.609	0.600	0.569	0.616	0.526	0.028
58	0.766	0.712	0.618	0.775	0.538	0.017
59	0.670	0.606	0.560	0.651	0.634	0.009
60	0.660	0.582	0.621	0.558	0.550	0.032
61	0.708	0.588	0.911	0.308	0.619	0.048
62	0.722	0.671	0.625	0.698	0.542	0.030
63	0.684	0.629	0.559	0.676	0.554	0.012
64	0.724	0.612	0.877	0.412	0.578	0.028
65	0.698	0.618	0.735	0.539	0.548	0.023
66	0.657	0.594	0.523	0.638	0.559	0.025
67	0.751	0.606	0.870	0.426	0.585	0.029
68	0.736	0.635	0.818	0.484	0.586	0.034
69	0.724	0.641	0.743	0.570	0.555	0.030
70	0.676	0.600	0.770	0.469	0.583	0.021
71	0.696	0.582	0.866	0.398	0.564	0.057
72	0.704	0.624	0.740	0.527	0.584	0.052
73	0.653	0.565	0.696	0.475	0.578	0.018
74	0.646	0.600	0.493	0.673	0.545	0.029
75	0.715	0.653	0.813	0.526	0.582	0.049
76	0.632	0.576	0.716	0.485	0.564	0.025
77	0.670	0.624	0.600	0.642	0.583	0.010
78	0.641	0.606	0.689	0.542	0.569	0.040
79	0.700	0.618	0.708	0.551	0.565	0.047
80	0.698	0.635	0.563	0.687	0.551	0.023
81	0.689	0.635	0.560	0.695	0.561	0.026
82	0.728	0.676	0.694	0.663	0.592	0.014

83	0.639	0.594	0.657	0.553	0.570	0.025
84	0.696	0.606	0.446	0.729	0.580	0.042
85	0.724	0.659	0.500	0.750	0.528	0.030
86	0.689	0.582	0.826	0.416	0.602	0.021
87	0.682	0.612	0.813	0.453	0.579	0.026
88	0.683	0.612	0.783	0.495	0.562	0.044
89	0.683	0.635	0.574	0.676	0.553	0.012
90	0.629	0.576	0.603	0.554	0.582	0.039
91	0.730	0.676	0.561	0.750	0.557	0.016
92	0.696	0.641	0.632	0.649	0.587	0.013
93	0.670	0.612	0.531	0.660	0.519	0.008
94	0.637	0.612	0.507	0.695	0.575	0.024
95	0.698	0.659	0.579	0.723	0.561	0.011
96	0.672	0.588	0.513	0.649	0.570	0.040
97	0.679	0.606	0.716	0.534	0.540	0.022
98	0.717	0.624	0.884	0.446	0.601	0.024
99	0.630	0.588	0.761	0.465	0.578	0.067
100	0.661	0.588	0.711	0.489	0.574	0.034
Average	0.685	0.615	0.662	0.581	0.564	0.028
Standard deviation	0.036	0.034	0.118	0.107	0.020	0.013
