# Supplementary material for: Urinary fatty acid biomarkers for prostate cancer detection
Source: PLoS One. 2024 Feb 9;19(2):e0297615. doi: 10.1371/journal.pone.0297615 (PMC10857612; doi:10.1371/journal.pone.0297615)
Supplement: S3 Table — The table presents the significant FA that were used to construct the diagnostic model, presented in order by their significance. (PDF) [file pone.0297615.s003.pdf]

S3 Table. FA selected in the final PCa diagnosis model. The table presents the significant FA that were used to construct the diagnostic model, presented in order by their significance.

FA Chain	Chemical Name	CAS #	Chemical Formula	P value[1]	PCa (n=334)	Control (n=232)	PCa %	Control %	Dominating Group
C4:1	Tiglic acid	80-59-1	$C_5H_8O_2$	1.074E-09	55	2	16.47	0.86	PCa
C10:0	n-Decanoic acid	334-48-5	$C_{10}H_{20}O_2$	4.764E-08	265	178	79.34	76.72	PCa
C9:0	Nonanoic acid	112-05-0	$C_9H_{18}O_2$	5.376E-06	225	143	67.37	61.64	PCa
C13:0	Tridecanoic acid	638-53-9	$C_{13}H_{26}O_2$	1.330E-05	116	48	34.73	20.69	PCa
C18:0	9-Octadecenoic acid	2027-47-6	$C_{18}H_{34}O_2$	1.446E-05	59	81	17.66	34.91	Control
C15:0	i-Propyl 14-methyl-pentadecanoate	1000336-62-4	$C_{19}H_{38}O_2$	1.557E-04	123	60	36.83	25.86	PCa
C22:0	Docosanoic acid, docosyl ester	17671-27-1	$C_{44}H_{88}O_2$	2.593E-04	114	55	34.13	23.71	PCa
C14:0	Tetradecanoic acid	544-63-8	$C_{14}H_{28}O_2$	3.710E-04	278	209	83.23	90.09	Control
C18:1	9-Octadecenoic acid, (E)-	112-79-8	$C_{18}H_{34}O_2$	3.819E-04	90	98	26.95	42.24	Control
C18:1	9-Octadecenoic acid (Z)-, methyl ester	112-62-9	$C_{19}H_{36}O_2$	6.867E-04	18	32	5.39	13.79	Control
C17:0	Heptadecanoic acid	506-12-7	$C_{17}H_{34}O_2$	1.144E-03	20	32	5.99	13.79	Control
C11:0	Undecanoic acid, 11-bromo-, undecyl ester	1000156-09-6	$C_{12}H_{23}BrO_2$	1.216E-03	21	2	6.29	0.86	PCa
C18:0	Methyl 2-hydroxystearate, TMS derivative	56196-58-8	$C_{22}H_{46}O_3Si$	0.004	43	14	12.87	6.03	PCa
C18:1	cis-9-Octadecenoic acid, propyl ester	1000405-15-0	$C_{21}H_{40}O_2$	0.007	0	5	0	2.16	Control
C5:1	4-Pentenoic acid, 2-methyl-, isobutyl ester	1000406-10-5	$C_{10}H_{18}O_2$	0.011	2	8	0.6	3.45	Control
C15:0	Pentadecanoic acid, 14-methyl-, methyl ester	5129-60-2	$C_{17}H_{34}O_2$	0.023	122	74	36.53	31.9	PCa
C16:0	Methyl 10-methyl-hexadecanoate	1000336-50-9	$C_{18}H_{36}O_2$	0.029	3	8	0.9	3.45	Control
C6:0	Hexanoic acid, 3-tetradecyl ester	1000279-29-7	$C_{20}H_{40}O_2$	0.032	3	8	0.9	3.45	Control
C18:0	Stearic acid hydrazide	4130-54-5	$C_{18}H_{38}N_2O$	0.035	19	5	5.69	2.16	PCa
C18:1	trans-13-Octadecenoic acid, methyl ester	1000333-61-3	$C_{19}H_{36}O_2$	0.041	6	11	1.8	4.74	Control

[1]P value obtained from Wilcoxon test of the FA ratio between prostate cancer and control groups.
