# Supplementary material for: Urinary fatty acid biomarkers for prostate cancer detection
Source: PLoS One. 2024 Feb 9;19(2):e0297615. doi: 10.1371/journal.pone.0297615 (PMC10857612; doi:10.1371/journal.pone.0297615)

S1 Figure. Histogram of the performance of the FA models in the 100 loop. The figure is a distribution of the performance (AUC) of the logistic regression models throughout the loop. The vertical line represents the mean (AUC= 0.685).

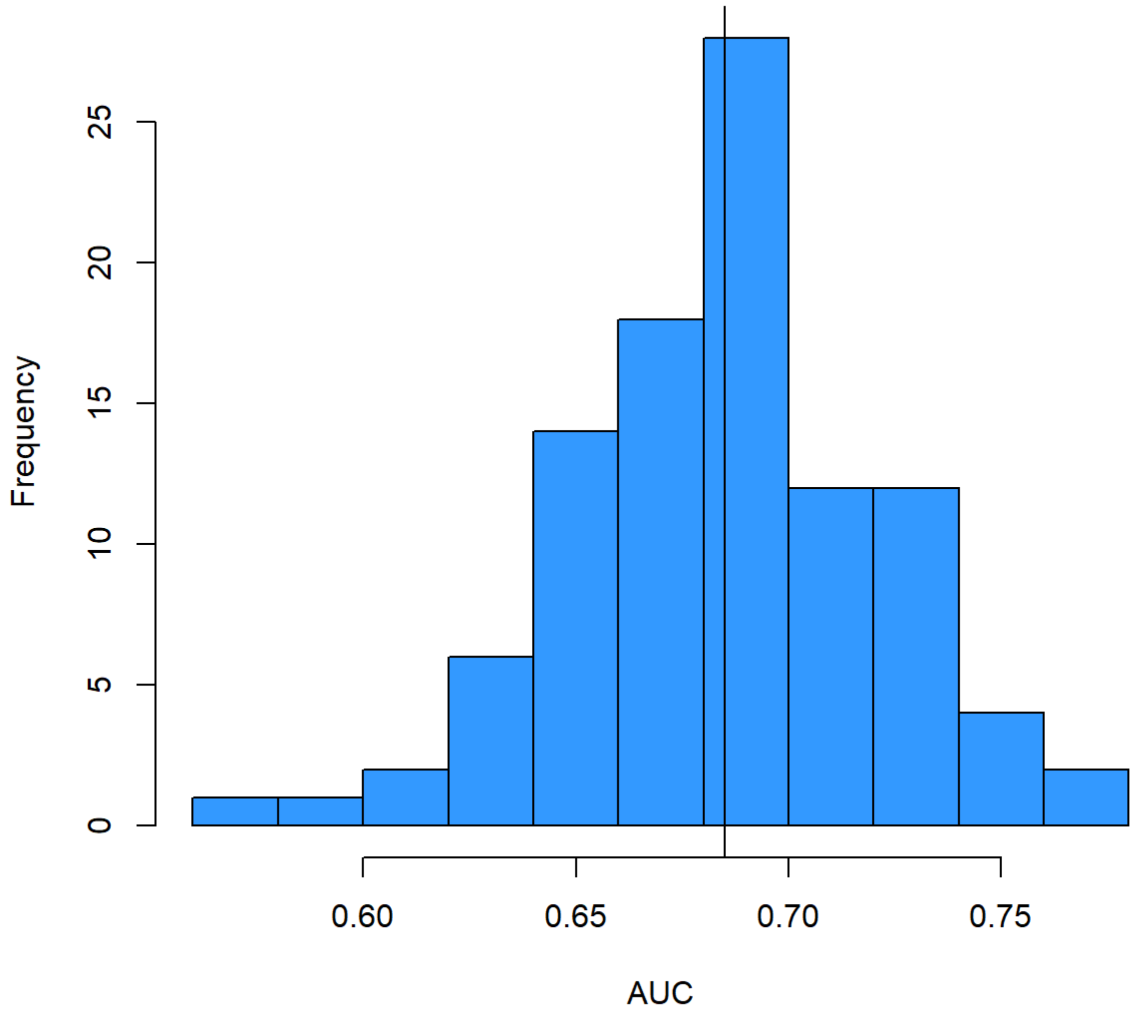

Supplement: S1 Fig — The figure is a distribution of the performance (AUC) of the logistic regression models throughout the loop. The vertical line represents the mean (AUC = 0.685). (PDF) [file pone.0297615.s004.pdf]
