# Supplementary material for: Urinary fatty acid biomarkers for prostate cancer detection
Source: PLoS One. 2024 Feb 9;19(2):e0297615. doi: 10.1371/journal.pone.0297615 (PMC10857612; doi:10.1371/journal.pone.0297615)

S2 Figure. Histogram of λ in the 100 loop. The figure is a distribution of the optimal tuning parameters for the logistic regression models throughout the loop. The vertical line represents the mean (λ= 0.028).

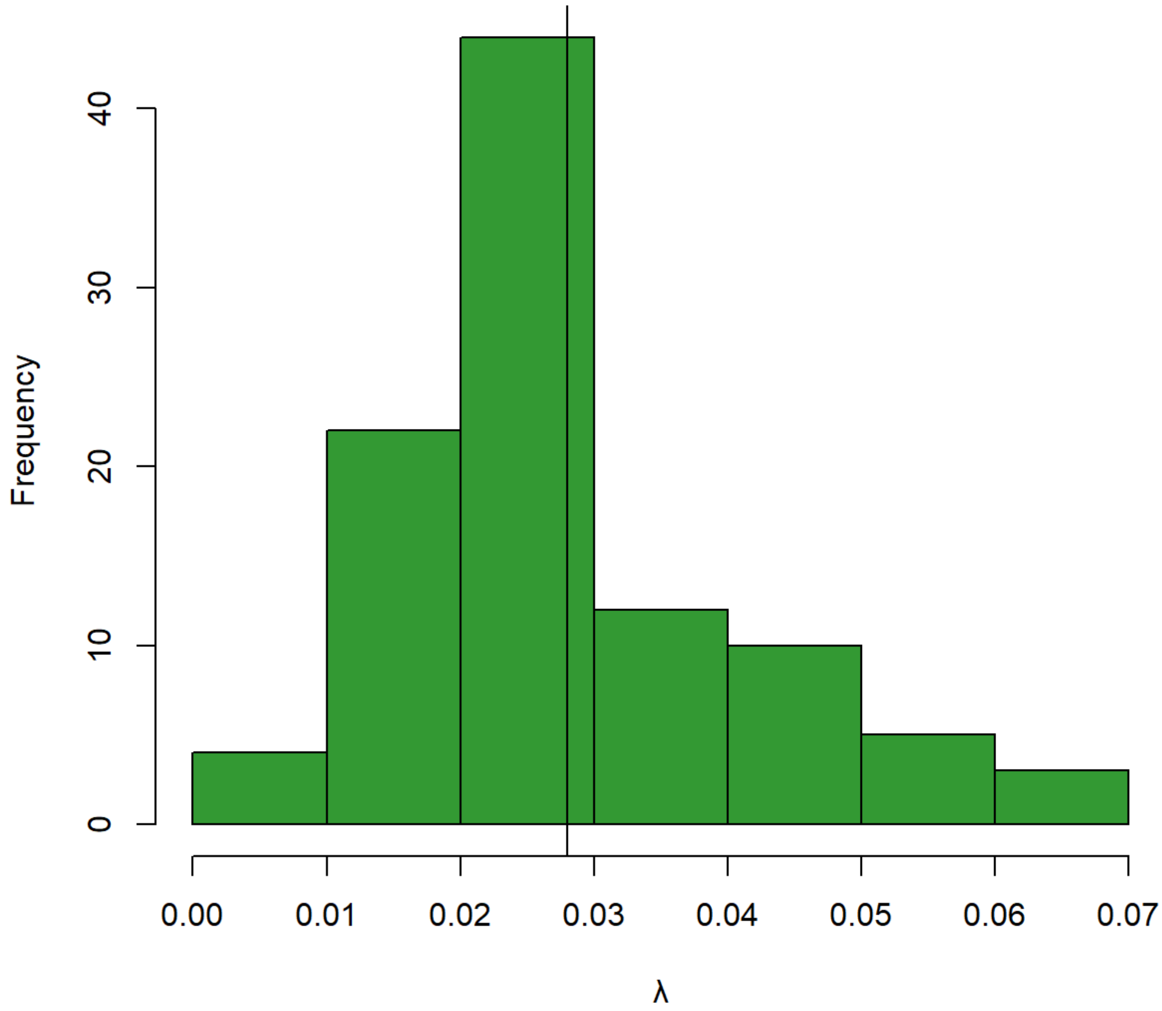

Supplement: S2 Fig — The figure is a distribution of the optimal tuning parameters for the logistic regression models throughout the loop. The vertical line represents the mean (λ = 0.028). (PDF) [file pone.0297615.s005.pdf]
